# Visualization of Single Polymer Chains with Atomic Force Microscopy: A Review

**DOI:** 10.3390/polym17101397

**Published:** 2025-05-19

**Authors:** Maria Pop, Otto Todor-Boer, Ioan Botiz

**Affiliations:** 1 Interdisciplinary Research Institute on Bio-Nano-Sciences, Babeș-Bolyai University, 400271 Cluj-Napoca, Romania; maria.botiz@ubbcluj.ro; 2 Department of Physics of Condensed Matter and Advanced Technologies, Faculty of Physics, Babeș-Bolyai University, 400084 Cluj-Napoca, Romania; 3 Research Institute for Analytical Instrumentation Subsidiary, National Institute for Research and Development of Optoelectronics Bucharest INOE 2000, 67 Donath Street, 400293 Cluj-Napoca, Romania; otto.todor@icia.ro

**Keywords:** single-chain atomic force microscopy, isolated polymer chains, thin films, molecular conformation

## Abstract

Single-chain atomic force microscopy has emerged as a powerful and highly specialized technique, enabling the direct observation and analysis of various isolated polymer chains at the nano and micro scales. This work reviews the most relevant experimental cases utilizing this technique, aiming to shine light on the understanding of the physical appearance of freshly synthesized polymer chains, reveal unique chain conformations and related transitions, decipher the processes of polymer crystallization and self-assembly, study the mechanisms of polymer adsorption and desorption, observe the formation of single-chain nanoparticles, and explore many other related phenomena.

## 1. Introduction

Polymers are macromolecular systems composed of numerous monomer units that are covalently bonded together. Since a monomer unit can exhibit virtually any chemical structure, polymers demonstrate a large variety of remarkable properties, including mechanical [1,2,3], biomedical [4,5,6,7,8,9], optoelectronic [10,11], thermoresponsive/conductive [12,13,14], and magnetic [15,16,17,18] characteristics, to name just a few. Consequently, scientists and engineers have leveraged these polymer properties over time to design and develop a plethora of practical applications [19,20,21,22,23,24,25,26,27,28]. These advancements have propelled not only major industries, such as adhesives [29,30], energy harvesting [31,32] and storage [33,34], aeronautics [35,36], automotives [13,37,38], and manufacturing and packaging [38,39,40,41]), but also specific interdisciplinary fields of research, including optoelectronics [42,43,44], detection [45,46], lithography [47,48,49], and advanced printing [50,51,52,53,54]), contributing to the betterment of society as a whole.

To continue developing novel polymeric applications with enhanced performance, it is essential to understand and master the relationship between the microstructure and its processing and properties. This relationship dictates the processing conditions necessary to generate an optimized polymeric microstructure with enhanced properties [11,55,56]. Furthermore, while a variety of processing methods can be utilized to manipulate the polymeric microstructure at both nano and micro scales [57], they all depend on several fundamental physical processes, including polymer phase separation [58,59,60,61], self-assembly [62,63,64,65], and crystallization [66,67,68,69]. These processes are contingent upon significant changes in the molecular conformation. Therefore, it is crucial to visualize the alterations in molecular conformations experienced by isolated polymer chains and to further comprehend the aforementioned processes, as well as other processes related to polymer synthesis, chemical structures, chain adsorption and desorption, and single-chain nanoparticle formation.

In recent decades, various methods have been developed to manipulate [70,71,72] and observe [73,74,75] single polymer chains on surfaces [74,76,77] and in solutions [78]. Among these techniques, atomic force microscopy (AFM) stands out as the primary method capable of both manipulating and observing single polymer chains in great detail. While the manipulation of polymer chains using AFM is exerted through specialized force–curve measurements [79,80,81], their direct observation is possible by recording AFM images and videos in various (fast) non-contact or contact modes. AFM is a highly effective technique widely used in materials science [22], biology [23], chemistry [24], and other fields. Since the invention of the scanning tunneling microscope, which provides information about the surface structure of matter at the atomic level [16,17], and the subsequent giant breakthrough that led to the development of AFM [19], the latter can now analyze almost any type of material [20] on a micrometer to nanometer scale (and in some cases, on a sub-nanometer scale) across a variety of environments [21].

In this work, we aim to review the most relevant scientific papers that report on the observation of single polymer chains using AFM. This review excludes molecular interactions studied through single-molecule AFM force spectroscopy as excellent reports on that topic can be found elsewhere [79,80,81]. Our primary goal is to highlight the advantages of single-chain AFM and assist the reader in visualizing the physical characteristics of polymer chains immediately after their synthesis or after being cast onto surfaces. We will explore their interactions and subsequent structure formation through self-assembly or crystallization, as well as their adsorption and desorption properties. Additionally, we will examine the formation of single-chain nanoparticles and track the scission and rupture of bonds, among other phenomena.

## 2. Utilization of Single-Chain AFM to Verify Synthesis and Confirm the Molecular Structure

**Figure 1:**
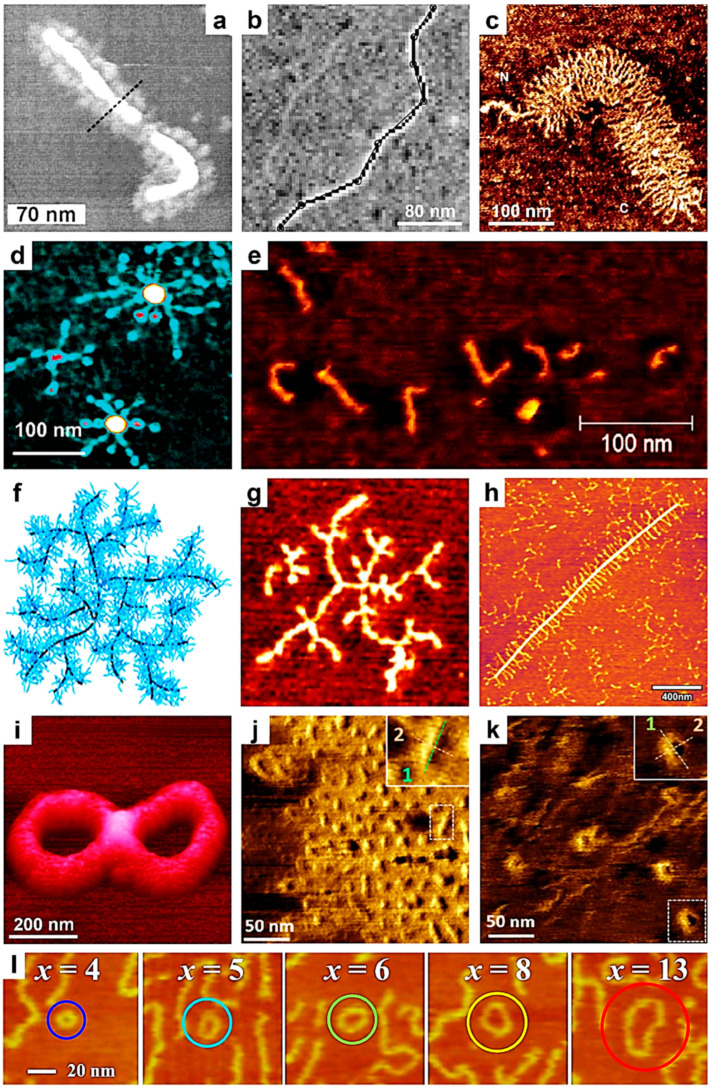
(**a**–**e**) AFM micrographs depicting a single molecule of a PBPEM-*g*-(P*n*BuA-*b*-PS) brush (**a**), a single 1054 bp DNA fragment deposited onto mica (**b**), a single fetal epiphyseal aggrecan monomer (**c**), a PS_7_-P2VP_7_ Pd-metallized unimolecular micelle (**d**), and single rigid rods of high-generation polynorbornene-based dendrons (**e**). (**f**,**g**) Schematic representation (**f**) and AFM visualization (**g**) of a molecular hyperbranched morphology obtained from a single B-SHBP molecule. The size of (**g**) is 80 × 80 nm^2^. (**h**) AFM micrograph illustrating rhamnogalacturonan I polysaccharides from the mucilage of *Arabidopsis* seeds. (**i**) AFM three-dimensional (3D) topographic representation of a PCEVE figure-of-eight molecular structure. (**j**,**k**) AFM images depicting linear (**j**) and globular objects (**k**) made of single grafted polymer chains containing selenol moieties. (**l**) Series of AFM micrographs (100 nm × 100 nm) depicting molecular cycles made of cyclic polymers and their fusions into multimers. Here, *x* represents the degree of the multimer. Reproduced from ref. [82] (**a**) [Copyright (2001), with permission from the American Chemical Society], ref. [83] (**b**) [Copyright (2001), with permission from Elsevier Science B.V.], ref. [85] (**c**) [Copyright (2003), with permission from Elsevier Inc.], ref. [89] (**d**) [Copyright (2003), with permission from the American Chemical Society], ref. [94] (**e**) [Copyright (2012), with permission from the American Chemical Society], ref. [96] (**f**,**g**) Published by The Royal Society of Chemistry, ref. [100] (**h**) [Copyright (2020), with permission from the American Chemical Society], ref. [103] (**i**) [Copyright (2009) WILEY-VCH Verlag GmbH & Co. KGaA (Weinheim) with permission from John Wiley and Sons], ref. [104] (**j**,**k**) [Copyright (2019) The Authors. Journal of Polymer Science Part A: Polymer Chemistry published by Wiley Periodicals, Inc. with permission from John Wiley and Sons], and ref. [105] (**l**) [Copyright (2019), with permission from the American Chemical Society].

Most conventional techniques used to characterize various macromolecules primarily focus on measuring the average properties of samples containing a multitude of these molecules. Such properties include the chemical composition, molecular weight, tacticity, dimensions, solubility, various thermal parameters, and more. In contrast, the AFM imaging technique provides a unique approach to analyze and investigate the structure of single macromolecular chains, particularly when these chains exhibit complex molecular architectures. Since the early 2000s, the AFM technique has emerged as a valuable tool for validating specific synthesis methods used to produce complex polymeric systems. Imaging single polymer chains with AFM allows researchers not only to observe and confirm the presence of poly(*n*-butyl acrylate-*block*-styrene) and poly(styrene-*block*-*n*-butyl acrylate) side chains, along with their globular tails (Figure 1a), in a brush of a poly(2-(2-bromopropionyloxy)-ethyl methacrylate)-*graft*-poly(*n*-butyl acrylate-*block*-styrene)/PBPEM-*g*-(P*n*BuA-*b*-PS) block copolymer (BCP) adopting extended wormlike conformations and synthesized via atom transfer radical polymerization (ATRP) [82], but also to accurately determine the length of macromolecules such as DNA (Figure 1b) [83]. Consequently, single-chain AFM measurements can be employed to validate certain methods for measuring DNA molecules (for instance, the Kulpa estimator) while invalidating others (such as the Freeman estimator) [83]. The transition from this capability to accurate molecular weight measurements by AFM was a short leap, achieved by Sheiko and coworkers, who determined the absolute molecular weight distribution of PBA brushes synthesized via ATRP by grafting *n*-butyl acrylate from a poly(2-(2-bromopropionyloxy)ethyl methacrylate) macroinitiator in 2003 [84]. This was achieved by performing AFM on a monolayer obtained through the Langmuir–Blodget (LB) technique and accurately determining the number of molecules per unit area. During the same period, Ng and coworkers utilized AFM to image single cartilage aggrecan macromolecules, successfully visualizing the conformation of individual aggrecan monomers and their constituent glycosaminoglycans (Figure 1c), as well as determining the corresponding chain trace length and end-to-end distances [85]. Later on, Wu and coworkers employed AFM to visualize, for the first time, single chains of oat *β*-glucans in extended conformations, determining their size, including contour length and end-to-end distance, and further computing the persistence length and weight-average molecular weight (*M_w_*) [86].

AFM is a delicate technique that requires patience and time. Measuring molecular weight using single-chain AFM is advisable only when other specialized methods are ineffective or cannot be utilized efficiently. Consequently, AFM has been employed to visualize the molecular structures of single polymer molecules with highly complex architectures, including linear/particle-coil copolymers [87], heteroarm star copolymers [88,89], arborescent polymers [90], dendronized polymers [91,92,93,94,95], and hyperbranched polymers [96]. In these instances, dedicated structural characterization methods were often limited or yielded less accurate results. For example, the single-chain AFM method was used efficiently not only to confirm the number of arms in the newly synthesized polystyrene-poly(2-vinylpyridine) (PS_7_-P2VP_7_) star copolymer, but also to visualize unimolecular micellar structures (Figure 1d) [88,89]. Moreover, by performing AFM studies on other single polymer chains of more complex architectures, such as those of dendronized polyferrocenylsilanes (PFSs) [91], dendronized polynorbornenes [92,94], dendronized conjugated di-BCPs [93], or various dendronized polymers based on diamines or diols [95], all corresponding molecular structures were clearly deciphered. These structures included spherical cocoons of single chains of dendronized PFS [91], single molecular wires comprising a regioregular backbone surrounded by bulky, insulating dendrons [93], single rigid rods of polynorbornenes containing high-generation dendrons (Figure 1e) [94], or even unimolecular tadpoles [92]. Obviously, the length and height of dendronized polymer chains could also be precisely determined by single-chain AFM [97].

More recently, bottlebrush-shaped segmented hyperbranched polymers (B-SHBPs) with a high grafting density were designed and synthesized by Zhang and coworkers by combining the self-condensing vinyl polymerization (SCVP) of hydroxyethyl methacrylate with various inimers and an accelerated copper-catalyzed azide-alkyne cycloaddition grafting-onto strategy [96]. By employing AFM on single B-SHBPs, they were able to design and visualize the single-molecule hyperbranched morphologies of B-SHBPs (Figure 1f,g) and further confirm a clear link between the reactivity of the polymerizable group in the inimers and the molecular SHBP morphology [96].

Polysaccharides represent another class of polymers characterized by complex molecular architectures. Their molecular structures are often elucidated by visualizing single [98,99] or occasionally a few [100] polysaccharide chains directly by AFM. For instance, as demonstrated by Ikeda and coworkers, AFM analysis of single chains of gum arabic and soybean polysaccharides revealed, for the first time, that while the former predominantly adopted linear structures, the latter exhibited a highly branched structure, with branches measuring from a few to several tens of nanometers in length [98]. The branched nature of polysaccharides was later confirmed by Round and coworkers, who conducted AFM on single pectin heteropolysaccharide chains. They emphasized that the eventual loss of various neutral sugars from the pectin samples had no significant impact on the size or branching density of the individual polymer chains [99] (more single-chain AFM studies on nanostructural modifications of pectin can be found elsewhere [101,102]). To add to these intriguing observations, we highlight a more recent study by Williams and coworkers, who performed AFM on a single (or possibly a few) polysaccharide rhamnogalacturonan I chain(s) extracted from the outer mucilage of *Arabidopsis* seeds. They demonstrated that regular and well-organized side chains were clearly present on these polymer chains (Figure 1h) [100].

Polymer cyclization, which involves chemical reactions that create ring structures within or from individual polymer chains, represents a scenario in which single-chain AFM offers significant analytical advantages. While Schappacher and Deffieux anticipated generating linear and cyclic molecular structures upon the cyclization of poly(chloroethyl vinyl ether) (PCEVE), their systematic AFM study of single PCEVE chains revealed the existence of other intriguing molecular structures, such as tadpole-shaped dimer molecules consisting of a ring and a linear chain, trefoil knot rings, catenanes, and figure-of-eight (Figure 1i) and dimer rings [103]. More recently, single-chain AFM has been employed not only to observe how the cyclization reaction induces the collapse of single linear polymer chains (Figure 1j), resulting in the formation of globular objects (Figure 1k) from non-folded wormlike brush structures [104], but also to evaluate, for the first time, the molecular cycles formed by cyclic polymers through ring-expansion-controlled radical polymerization reactions and further observe their fusion into multimers (Figure 1l) [105].

Finally, it is important to note that single-chain AFM can be further utilized to visualize the structure of single isotactic poly(methyl methacrylate) (*it*-PMMA) chains, particularly when they are end-capped with C_60_ fullerene [106], or to monitor the dynamic motions and structural changes of DNA biomolecules within DNA origami nanostructures [107]. The latter example aims to elucidate the physical properties of individual DNA molecules and their reaction mechanisms. A more comprehensive list of the benefits resulting from the use of single-chain AFM can be found in Table 1.

## 3. Using Single-Chain AFM to Monitor Chain Conformation and Conformational Transitions

In this section, we will demonstrate that the AFM technique can be employed not only to observe the conformation of single polymer chains but also to monitor various conformational transitions that these chains may undergo under different laboratory conditions. Indeed, in their early report in 1992 [108], Hansma and coworkers successfully imaged single plasmid DNA molecules deposited on mica using specially sharpened AFM tips. Since then, other single-chain AFM experiments on DNA have shown that when binding various drug ligands such as doxorubicin or ethidium bromide to DNA molecules, the latter may experience a change in flexibility and an increase in length [137].

**Figure 2:**
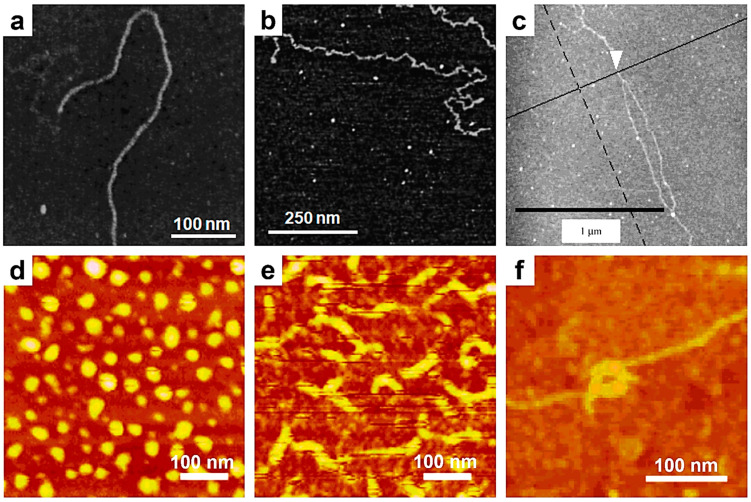
(**a**,**b**) AFM micrographs depicting single rigid (**a**) and flexible (**b**) succinoglycan chains. (**c**) AFM image illustrating a xanthan chain displaying a double helical conformation, which occurred upon its renaturation. (**d**–**f**) AFM micrographs showing dots (**d**) and rigid rods (**e**) generated from polysilane single chains, as well as toroid supramolecular structures (**f**) generated from at least two polysilane chains. Reproduced from ref. [138] (**a**,**b**) [Copyright (2000), with permission from the American Chemical Society], ref. [139] (**c**) [Copyright (2001), with permission from the American Chemical Society], and ref. [111] (**d**–**f**) [Copyright (2003), with permission from the American Chemical Society].

In the meantime, the single-chain AFM technique has significantly expanded its range of applicability to various natural and synthetic polymers. For instance, Kumaki and coworkers visualized the conformation of single polystyrene-*block*-poly(methyl methacrylate) (PS-*b*-PMMA) di-BCP chains deposited on mica using AFM as early as 1996 [109]. Subsequently, AFM was employed to observe single-chain molecular conformations of more complex polymeric systems, such as succinoglycan [138] and xanthan [139] polysaccharides. The former was employed by Balnois and coworkers to differentiate between rigid (Figure 2a) and flexible (Figure 2b) succinoglycan chains [138]. In contrast, xanthan was prepared by Camesano and coworkers in very dilute solutions and further deposited on mica, aiming to probe and characterize not only the representative single-chain conformations of xanthan but also its secondary helical structure (Figure 2c), which occurs upon renaturation in water [139]. In the case of xanthan, various chain parameters, including the number–average contour length and persistence length, were precisely measured and compiled under different experimental conditions. More recently, single chains of xanthan were again observed, using AFM with Arrow UHF-AuD probes, clarifying that each helix is formed by a double strand and demonstrating that helical conformations may be deformed upon adsorption from aqueous solutions onto charged mica [110]. We conclude this discussion on polysaccharides by mentioning the case of bacterial curdlan [140]. Specifically, using AFM, Jin and coworkers demonstrated that curdlan single chains adopt a flexible, disordered conformation when deposited from a low-concentration dimethyl sulfoxide aqueous solution [140]. However, when deposited from more concentrated solutions, curdlan chains exhibited more rigid conformations and tended to aggregate into network-like structures.

Other instances in which the AFM technique was employed to illustrate single-chain conformations include semiconducting polysilane [111], poly(phenyl isocyanide)s containing *L*-alanine or *L*-lactic acid residues with long *n*-decyl chains as pendant groups [141], and miscible polymer blends composed of PMMA and poly(*n*-nonyl acrylate) (PNA) [142]. In the case of poly(phenyl isocyanide)s, long single chains were distinctly imaged while packed in amorphous 2D films prepared using the LB method [141]. In the other two cases, single-chain AFM was used to directly observe the conformations of single polymer chains, grafted either through a conventional grafting approach [111] or by solubilizing the polymer chain of interest within a monolayer made of a different type of polymer [142]. This method allowed for a clear distinction between dots displaying a globular conformation (Figure 2d) and ropes displaying a rigid rod-like conformation (Figure 2e) formed by single polysilane chains. Furthermore, when a greater number of polysilane chains were grafted per unit area, AFM revealed that these chains interacted and formed more complex supramolecular entities, such as octopus and toroid (Figure 2f) formations [111].

**Figure 3:**
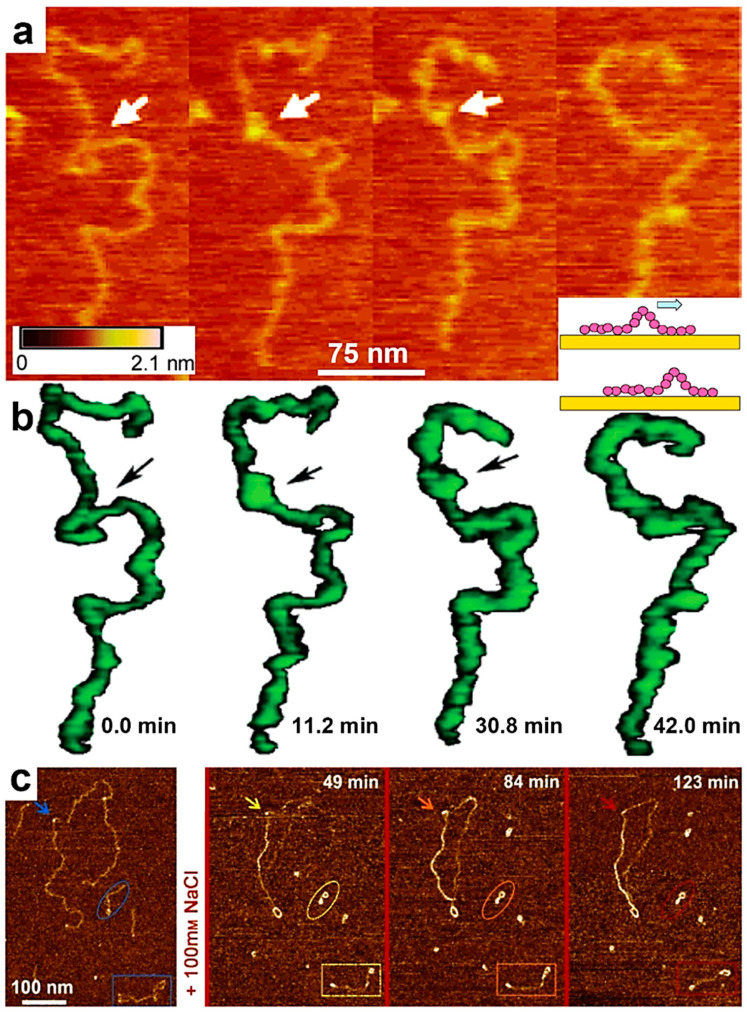
(**a**) Series of AFM micrographs displaying the movements of a single *it*-PMMA chain on mica, which mimic snakes/caterpillars. (**b**) Chain images inferred from the AFM images shown in (**a**). The inset represents a schematic illustration of PMMA chain movements on a mica substrate. (**c**) Time-lapse AFM micrographs illustrating the coil-to-helix transition of a single iota-Na-carrageenan chain on mica previously immersed in MilliQ water and at three different time points after the replacement of this environment with a NaCl aqueous solution. Reproduced from ref. [115] (**a**,**b**) [Copyright (2006), with permission from the American Chemical Society], and ref. [113] (**c**) [Copyright (2014) The authors. WILEY-VCH Verlag GmbH & Co. KGaA (Weinheim) with permission from John Wiley and Sons].

In the second part of this section, we will further summarize several prominent examples in which the AFM was successfully used to monitor the conformational transitions experienced by single chains of synthetic PS-*b*-PMMA [112], *it*-PMMA [115], polyelectrolytes [114,143,144], natural polysaccharides [113], and DNA [107]. In the early to mid-2000s, conformational changes at the level of individual PS-*b*-PMMA and *it*-PMMA chains, as observed by AFM, were reported by Kumaki and coworkers. They have shown that depositing single PS-*b*-PMMA molecules at low surface pressures resulted in aggregated PS particles, each connected to a single non-aggregated PMMA chain. The PMMA chains subsequently aggregated, forming a condensed monolayer around each PS particle. This overall conformation was shown to transition once again into PS particles with emerging PMMA chains, the latter adopting an expanded and elongated coil conformation when the deposited film was exposed to highly humid air for one day [112]. Furthermore, single *it*-PMMA flexible chains isolated on a mica substrate were visualized by AFM and shown to experience interesting water-dependent “reptational” movements, i.e., chains moved along their long molecular axis on the mica surface in a manner reminiscent of snakes and caterpillars (Figure 3a,b) [115].

Other single-chain conformational transitions from coiled conformations have been reported in polyelectrolytes such as poly(2-vinylpyridine) and poly(methacryloyloxyethyl dimethylbenzylammonium chloride) (PMB) [114,143]. For example, by employing single-chain AFM, researchers have demonstrated that a single flexible PMB molecule can undergo a series of conformational transitions, shifting from an elongated coil to transient pearl necklace–globule conformations, and finally to a globule conformation when deposited on solid mica from a poor solvent while experiencing an increase in the ionic strength of the primal solution [114].

We conclude this section by discussing two examples of natural polymeric systems that have been shown to undergo conformational transitions using single-chain AFM. In the first example, high-speed single-chain AFM was used to describe the dynamic motions of individual DNA molecules and their structural changes within DNA origami surface relief structures [107]. This approach has contributed to the development of innovative methods for visualizing the movements of single biomolecules in real time and at molecular resolution [107]. The second example depicts the structural conformations of an anionic iota-Na-carrageenan polysaccharide [113]. It has been demonstrated that this polysaccharide undergoes a coil-to-helix transition in the presence of a monovalent salt (Figure 3c). During this transition, its persistence length increased from approximately 22 nm in the random coil state to over 26 nm in the newly adopted, ordered, and yet more rigid helical conformation, clearly demonstrating the intramolecular formation of unimeric helices from single polymer chains. A summary of the benefits arising from the use of single-chain AFM can be found in Table 1.

## 4. Understanding Crystallization and Self-Assembly Processes by Using Single-Chain AFM

Visualizing single-chain polymeric structures is crucial for understanding physical processes such as polymer crystallization and self-assembly. Although single-chain observations can be performed using the AFM technique, it remains highly challenging to precisely monitor soft polymeric chains and their conformational changes at the molecular level. Despite these difficulties, AFM has successfully demonstrated the observation of various polymeric single-chain structures and their crystallization in many 2D films when appropriate scanning conditions are employed [145]. While earlier studies concentrated on deciphering the unfolding of chains incorporated in single crystals that were generated from very long alkanes (PE) or polyethylene [146], later studies have employed single-chain AFM to observe isolated linear chains of ultrahigh-molecular-weight PE, along with their transitions from random-coil to rod-like crystalline conformations [147].

**Figure 4:**
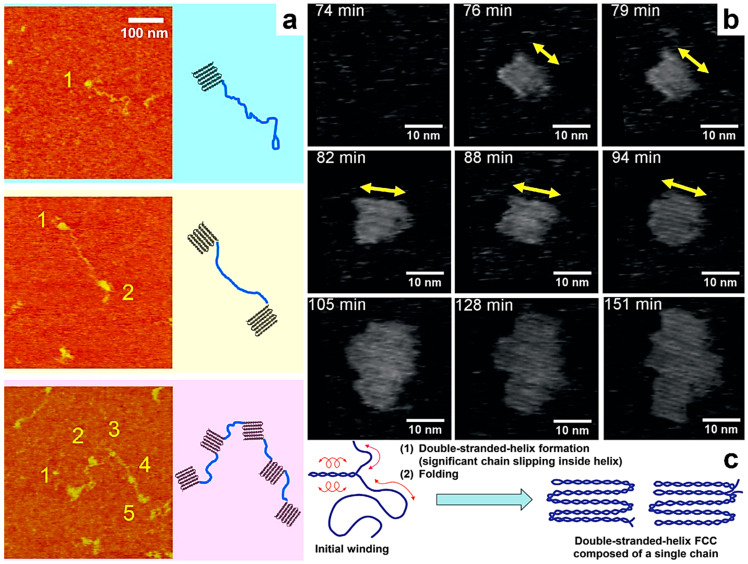
(**a**) Series of AFM micrographs (**left**) and schematics (**right**) depicting various crystalline structures of single *it*-PMMA chains: chains crystallized at one end (**top**), at both ends (**middle**), and at five points (both ends and three in the middle of the chain), similar to a necklace (**bottom**). (**b**) Time-lapse AFM phase micrographs showing an *it*-PMMA crystal that formed at 76 min and had a size comparable to that of a single folded-chain crystal until 94 min, when it started to grow to a multichain crystal containing around 5 chains. (**c**) Schematic illustration of the crystallization of unimolecular folded-chain crystals: an *it*-PMMA chain suffers intramolecular intertwining and generates a double-stranded helix inside the chain. Later on, the entire chain forms a double-stranded helix and further folds to generate a unimolecular folded-chain crystal. Reproduced from ref. [116] (**a**) [Copyright (2015), with permission from the American Chemical Society], ref. [118] (**b**,**c**) [Copyright (2021) WILEY-VCH GmbH, with permission from John Wiley and Sons].

More recently, Kumaki and coworkers have begun to further investigate the crystallization behavior of single polymeric chains by observing isolated *it*-PMMA single chains [116]. By employing time-lapse single-chain AFM (using standard silicon cantilevers) and solubilizing high-molecular-weight compounds in a monolayer of a non-crystallizable, low-molecular-weight *it*-PMMA oligomer, the authors have visualized, for the first time, the crystallization behavior of an isolated *it*-PMMA chain at the molecular level. They have also inferred from statistical observations how isolated *it*-PMMA chains transition to unimolecular crystals composed of small crystallites interconnected by an amorphous chain necklace (Figure 4a) [116].

Later, the same research group advanced the understanding of single-chain crystallization by observing, in situ and in real time, not only the folding of isolated *it*-PMMA chains during their crystallization [117] but also the formation of folded-chain crystals from these single *it*-PMMA chains [118]. These unimolecular crystals, which are the smallest size, i.e., single-chain crystals, were shown to further grow into larger multichain crystals through the one-by-one incorporation of additional single chains (Figure 4b; standard AFM cantilevers were utilized) [118]. Moreover, both unimolecular and multichain crystals were composed of folded double-stranded helices, each formed from a single *it*-PMMA chain that underwent intramolecular intertwining (Figure 4c) [118]. This series of studies on the folded-chain crystallization of *it*-PMMA was recently expanded by conducting single-chain AFM measurements, both in situ and in real time. This approach enabled, for the first time, the visualization of the stem-level crystallization of a single isolated *it*-PMMA chain into a folded-chain crystal [119]. Yet another recent study performed on poly(ethylene oxide) (PEO) by combining AFM with single-molecule force spectroscopy further emphasized that polymer crystallization starts with the folding of individual PEO chains into 8–9 nm large cluster-resembling structures. The latter further interact to generate larger crystalline structures [148]. Nonetheless, it is worth mentioning that the direct observation of crystallization at the molecular level of folded chains in real time remains highly challenging and extremely limited at the moment.

Similar to crystallization, single-chain AFM can also be employed to study and eventually decipher the process of polymer self-assembly. In 1997, McIntire and Brant had already employed single-chain AFM and imaged isolated molecules of various biopolymers, such as scleroglucan, xanthan, gellan, collagen, or *k*-carrageenan, as well as their assembled superstructures [120]. They found that the assembly of such biopolymers into supramolecular (fibrillar) entities could be favored by establishing control over molecular weight distributions or through the addition of low-molecular-weight salts. Furthermore, *k*-carrageenan was studied using single-chain AFM by Ikeda and coworkers, who visualized non-aggregated and aggregated helices of this system on mica. They demonstrated that in the presence of salt, *k*-carrageenan generated rigid, branched, rod-like aggregates made of double helices [149]. Other natural polymeric systems, such as cellulose-based chains, were assembled from solutions of different salt concentrations on highly oriented pyrolytic graphite and further studied using single-chain AFM [121]. While it was already shown that the alignment of rigid molecular rods made of single cellulose chains proceeds through a specific attraction between cellulose and oriented graphite [150], drastic conformational changes from aligned cellulose single chains to globular aggregates were reported when salt concentrations were increased [121].

**Figure 5:**
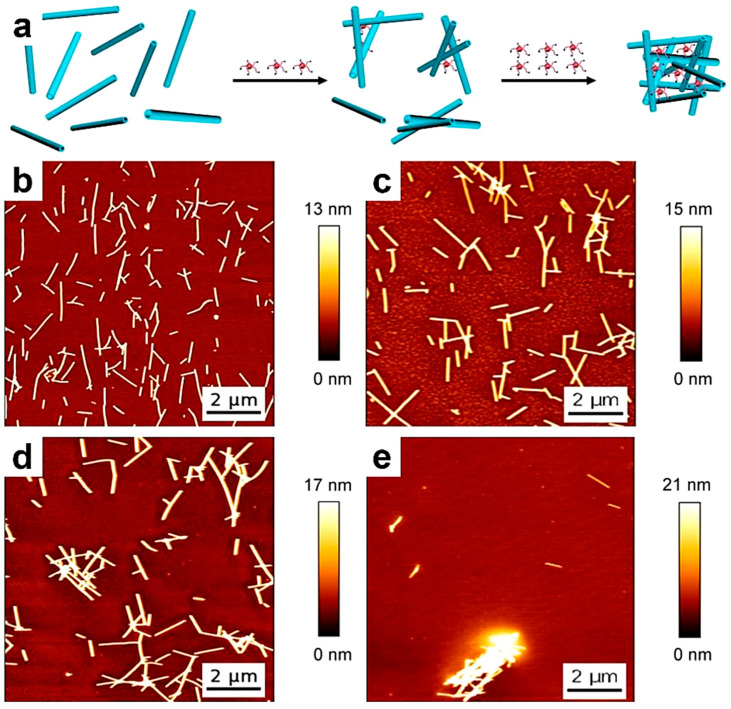
(**a**) Schematic representation of the effect of tannin concentrations on the aggregation of TMV particles. (**b**–**e**) AFM topography micrographs depicting TMV particles without (**b**) and with 0.09 mg/mL (**c**), 0.9 mg/mL (**d**), and 22.5 mg/mL (**e**) of tannin. Reproduced from ref. [122] (**a**–**e**) Published by The Royal Society of Chemistry.

More recently, single-chain AFM was used to study the assembly of two other natural polymeric systems: fibrin [151] and polyphenolic tannin [122]. While fibrin molecules have been reported to generate supramolecular fibers of different sizes and densities, and experienced decreased molecular packing with an increase in their diameter [151], tannin molecules were combined with the tobacco mosaic virus (TMV) to illustrate the effect of tannin on the aggregation mechanism of TMV particles and understand their antiviral mechanism [122]. In this latter case, while TMV particles exhibited no strong TMV–TMV interactions in the absence of tannin, an increase in the concentration of tannin molecules led to greater aggregation of TMV particles through the tannin–TMV hydrogen bonding and hydrophobic interactions (for example, AFM studies have shown that increasing the tannin concentration from 0.09 mg/mL to 0.9 mg/mL generated TMV aggregates of increased size that contained an average of six and ten TMV particles, respectively) (Figure 5). It is this aggregation that reduces the chance of TMV particles entering the cells of plants [122]. A similar approach to generate protein-based supramolecular assemblies was taken very recently by Hlushko and coworkers, who employed single-chain AFM to visualize not only isolated chains of synthetic poly[di(carboxylatophenoxy)phosphazene] (PCPP) macromolecule in their vitrified state but also supramolecular assemblies generated upon their assembly with antigenic proteins such as bovine serum albumin and hen egg lysozyme [123]. The authors discovered that certain proteins have the ability to attach to the PCPP chains and spontaneously generate single-chain structures that resemble compact spherical complexes or stiffened coils (for instance, the stiffening of PCPP chains upon bovine serum albumin binding was revealed by determining their persistence length from AFM measurements) [123].

Furthermore, the self-assembly of synthetic polymeric systems, such as dendronized polyphenylacetylene (PPA) [152], folded BCPs comprising a cylindrical brush and a single-chain nanoparticle [153], tri-BCP bottlebrushes (PS-PD-PS) made by grafting polynorbornene backbones with PS and poly[2-(dimethylamino)ethyl methacrylate] [154], or polythiophene [155], can also be monitored by taking single-chain AFM measurements. Reported experiments have revealed puzzling transitions from individual PPA chains adopting an oblate cylindrical conformation to (periodic) assemblies, such as large domains exhibiting a 2D nematic order [152], from dumbbell-shaped single-chain conformations to interesting discrete oligomeric aggregates of tri-BCP bottlebrushes displaying a well-defined structure and including linear and cyclic dimers, tadpole-like trimers, or higher oligomers [154], and from individual units to single strands of polythiophene [155].

## 5. Revealing Polymer Adsorption and Desorption Properties with Single-Chain AFM

The adsorption of various polymer chains onto surfaces is crucial in numerous industrial applications, including surface fouling, and plays pivotal roles in flocculation and coating processes, pattern recognition, drug delivery, tissue engineering, the design of novel nanocomposites, and biosensing, to name just a few. In 2000, Gunning and coworkers conducted single-chain AFM on a cell wall polysaccharide physiosorbed on mica, and observed the motion of this polymeric chain in an aqueous buffer solution [124]. This motion appears to result from the repetitive desorption and re-adsorption of molecular chain segments from and to mica (i.e., changes in the binding of a single polysaccharide to a charged substrate), as indicated by the existence of loops, trains, and tails [124]. Shortly after, the authors further described, in great detail, the dynamics of the adsorption and desorption of polysaccharide chains at the single-chain level, demonstrating not only that the three hypothesized classical states of loops, trains, and tails could be followed in time, but also that loops appeared as a result of a local conformational change suffered by the polysaccharide chain [156].

In the next two years, after acknowledging the importance of the adsorption of polyelectrolytes on solid substrates [144], Roiter and coworkers reported single-chain AFM studies on polyelectrolyte chains, such as P2VP [125] and PMB [126], absorbed on mica. They have shown that while adsorbed single P2VP chains transitioned from an extended coil conformation to a compressed globule with a decrease in the ionization degree [125], the adsorbed PMB chains, when exposed to increased concentrations of Na_3_PO_4_ salts, suffered sharp conformational transitions from an extended coil-like conformation to a pearl necklace-like globular conformation [126]. Most probably, this transition was due to stronger van der Waals interactions with mica. Later on, the authors further provided evidence on the behavior of P2VP single chains adsorbed at a solid–liquid interface in dilute, moderate, and high salt concentration regimes, demonstrating that the corresponding chain conformation adopted by P2VP chains was represented by extended coils, shrunken coils, and re-extended coils, respectively [157].

Other polymeric systems involved in studies of adsorption and desorption on solid surfaces include poly(styrenesulfonate) (PSS) [127] and commercial Nafion [128]. While in the former case, PSS single chains adsorbed on mica were shown to adopt a wormlike coil conformation (this conformation changed to a morphology of circular patches when PSS chains were co-adsorbed along with hexadecyltrimethylammonium bromide molecules at an air–water interface) [127], in the latter case, autoclaved Nafion single chains were adsorbed on both mica and graphite and shown to adopt an electrostatically stabilized conformation resembling compact globules [128]. Note that tapping-mode silicon nitride probes displaying a spring constant of 0.32 N/m, a resonance frequency of ∼9 kHz, and a radius of curvature of ∼10 nm were used.

**Figure 6:**
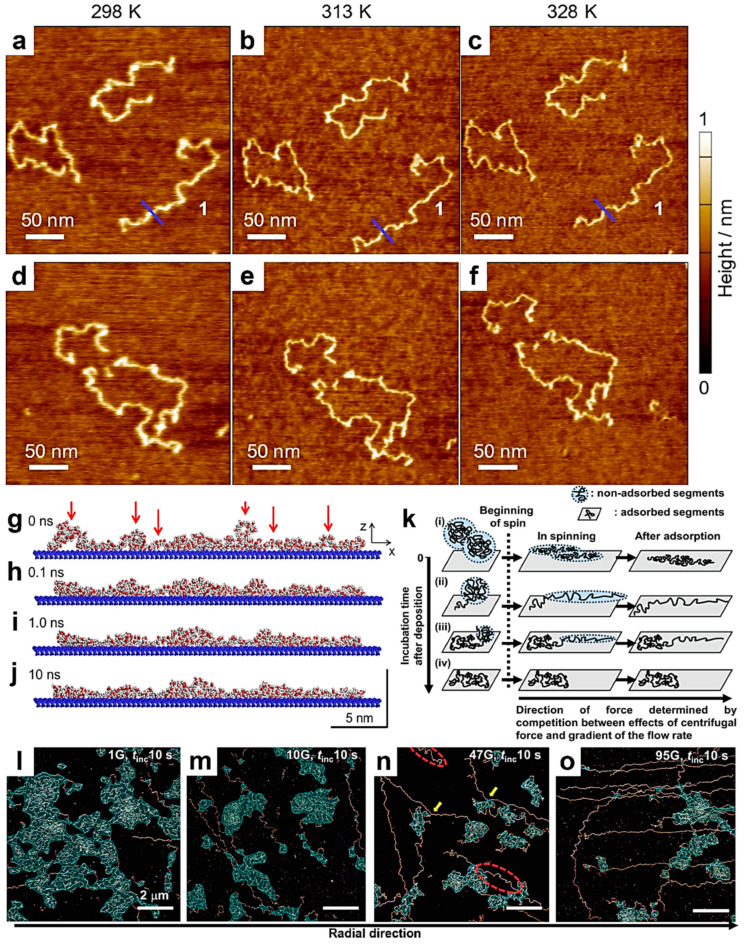
(**a**–**f**) AFM height micrographs depicting PMMA single chains adsorbed on mica at 298 K (**a**,**d**), 313 K (**b**,**e**), and 328 K (**c**,**f**). (**g**–**j**) Time-lapse snapshots (projected to the *zx* plane) of a PMMA single chain adsorbed on a solid substrate at 328 K after 0 ns (**g**), 0.1 ns (**h**), 1.0 ns (**i**), and 10 ns (**j**). Here, while carbon, hydrogen, and oxygen atoms were colored with gray, white, and red, respectively, the solid substrate was colored in blue. Red arrows indicate chain segments in loops that have the tendency to establish contacts with the substrate. (**k**) Schematics depicting the effect of spinning on the adsorption of DNA molecules on mica. (**l**–**o**) AFM micrographs emphasizing the DNA chains deposited on mica by spin-casting using a relative centrifugal force of 1 G (**l**), 10 G (**m**), 47 G (**n**), and 95 G (**o**). Yellow arrows indicate changes in the orientation direction within a single DNA chain. Red dotted ellipses denote kinks in stretched DNA chains. All samples were prepared using an incubation time of 10 s. Areas shaded in blue designate 2D random coils. The scale bar for all images is 2 µm. Reproduced from ref. [129] (**a**–**j**) [Copyright (2020) The author(s), with permission from Springer Nature] and ref. [130] (**k**–**o**) [Copyright (2024) The author(s), under exclusive license to The Society of Polymer Science, with permission from Springer Nature].

More recent studies reported in the literature have focused on the adsorption of single chains of PMMA [129] and DNA [130]. For example, Oda and coworkers have adsorbed single chains of PMMA on mica and followed (using single-chain AFM combined with a molecular dynamics simulation) their morphological transition induced by an increase in temperature. Their results have shown that PMMA chains become rigid as they approach a pseudo-equilibrium state by changing their local conformation from loops to trains (Figure 6a–j) while maintaining their relative positions on mica as almost unchanged [129]. Instead, Morimitsu and coworkers adsorbed DNA molecules on mica by employing a spin-casting technique while varying parameters, such as the incubation time (i.e., the time that passed between the deposition of DNA-containing droplets and the start of spinning) and the strength of the relative centrifugal forces (Figure 6k) [130]. Their single-chain AFM measurements revealed that while longer incubation times favored more DNA chains to undergo transitions from 2D random coils to stretched conformations, shorter incubation times led to the adsorption of many DNA chains in a stretched conformation, often displaying conformational kinks. Moreover, the appearance of competition between the effects induced by the centrifugal force and the flow rate gradient dictated the orientations of the stretched DNA chains on mica (Figure 6l–o) [130].

## 6. Employing AFM to Monitor the Generation of Single-Chain Nanoparticles

**Figure 7:**
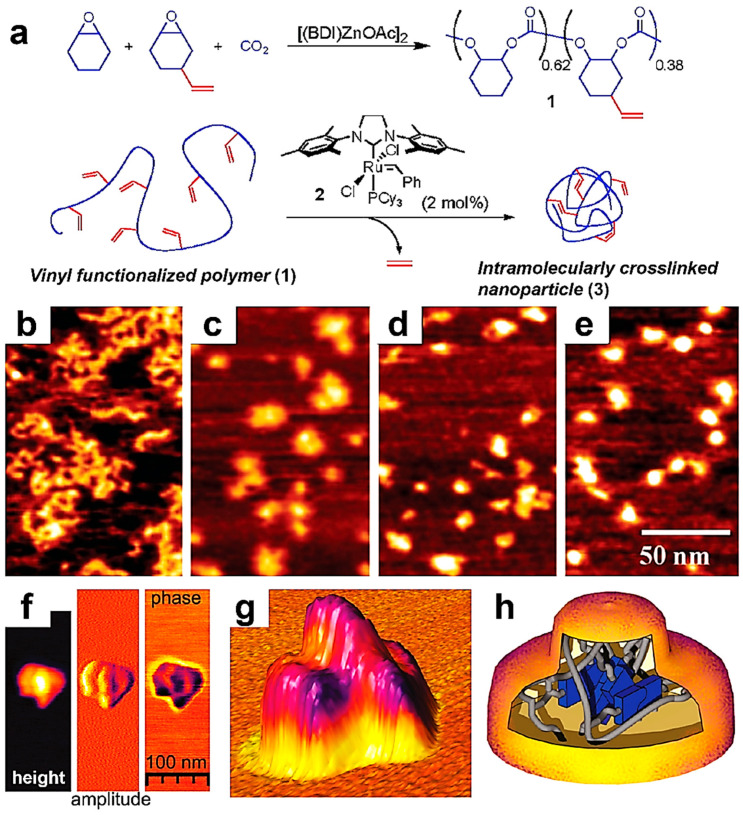
(**a**) Schematics representing the synthesis of alkene cross-linked polycarbonate SCPNPs. (**b**–**e**) AFM height micrographs depicting polycarbonate single chains (**b**) and SCPNPs obtained after 0.25 h (**c**), 0.5 h (**d**), and 4 h (**e**) of cross-linking. The scale bar for all images is 50 nm. (**f**) AFM height, amplitude, and phase micrographs illustrating individual SCPNPs made of UPy-functionalized PMMA and raised cores. (**g**) Three-dimensional AFM height micrograph showing the SCPNP presented in (**f**). (**h**) Cartoon portraying a possible morphology of the SCPNP shown in (**f**,**g**) and comprising an UPy-urethane rich core (blue) embedded in a network of PMMA. Reproduced from ref. [135] (**a**–**e**) [Copyright (2007), with permission from the American Chemical Society], and ref. [136] (**f**–**h**) [Copyright (2010), with permission from the American Chemical Society].

Single-chain polymeric nanoparticles (SCPNPs) comprise single polymer chains that underwent partial/total folding, collapse, or cross-linking. They became an attractive topic of research due to their interesting advantages, including a small (from 1.5 to 20 nm) yet highly controlled size, the possibility of generating well-defined (directional) molecular conformations that further allow customized arrangements of various (multi)functional groups, and the availability of a wide range of chemical compositions [158,159], to name just a few. Thus, SCPNPs started to be used in various applications, including sensing, catalysis, nanomedicine, and many more. Here, while we will not discuss the details of the types of methods used to generate SCPNPs or determine their functionalities (these details are available in comprehensive studies published elsewhere [158,159,160]), we aim to emphasize several cases where single-chain AFM demonstrates its key role in revealing peculiar information regarding the formation of SCPNPs that could not be retrieved using other, more conventional methods. These cases include monitoring cross-linking reactions that generate SCPNPs [135], observing chain folding [136], and characterizing SCPNPs [160,161]. Thus, it is clear that single-chain AFM is a reliable tool to observe, for instance, the synthesis of SCPNPs through the combination of covalent and non-covalent intrachain cross-linking [160], as well as the conversion of linear polycarbonates into SCPNPs of well-controlled sizes through a covalent cross-linking reaction (Figure 7a) [135]. This was possible through the visualization of single polycarbonate chains at different stages of the cross-linking reaction and their transition from extended molecules that spread over a large area on mica to increasingly compact molecules that cover progressively smaller areas [135], as depicted in Figure 7b–e.

Moreover, high-resolution single-chain AFM has further demonstrated its unique capabilities in providing essential details on the intramolecular collapse of single polymeric chains of PMMA functionalized with an *o*-nitrobenzyl-protected 2-ureidopyrimidinone (UPy) pendant moiety (this moiety was linked to PMMA through a urethane group), and their transition to individual SCPNPs [136]. The latter were shown to possess a complex geometry, displaying a raised center with dimensions almost matching the size of the UPy–urethane dimer (Figure 7f,g). Furthermore, AFM results showed that this core was different from the rest of the individual SCPNPs in both height and phase, pointing towards a separate internal phase mainly containing UPy–urethane dimers (Figure 7h) [136]. Finally, it is worth mentioning that AFM was recently also employed to image and confirm the generation of 12 nm large single-chain polymer dots made of donor–acceptor–donor alkoxy thiophene–benzobisthiadiazole-based conjugated polymers [162]. Such single-chain dots possess emissive properties and are regarded as promising candidate structures for bioimaging applications.

## 7. Using Single-Chain AFM to Determine Chain Stiffness and Probe Chemical Contrasting

The stiffness of polymer (DNA) chains was already tackled by single-chain AFM in the beautiful 1998 study of Rivetti and coworkers, who extended the wormlike chain model from long, straight polymer chains to polymers experiencing sections of dissimilar flexibility (for example, single/coplanar/non-coplanar bends) or unalike persistent lengths, revealing, at the end, a complete conformational analysis of the flexibility of single- and double-stranded DNA molecules and demonstrating the possibility of clearly determining the magnitude of DNA bends [163]. Several years later, Maurstad and coworkers employed single-chain AFM to develop a comprehensive analysis on semiflexible polyanions (e.g., alginate, acetan, xanthan, and circular plasmid DNA) of rather compacted conformation, with the goal of deciphering the impact of chain stiffness on various morphologies displayed by polyelectrolyte complexes [132]. They discovered that the complexation of polyanions with chitosan forced more or less polyanion molecules to generate interesting torus-like and persistence-length-dependent morphologies, with additional rod-like and racquet morphologies also being reported forming under specific temperatures and molecular weights (tapping-mode silicon nitride cantilevers with nominal spring constants of 20–100 N/m and nominal resonance frequencies of 200–400 kHz were utilized) [132]. Controlled environments were further used to expose samples of poly(isocyanodipeptides) (PICs) adsorbed on mica to a specific amount of humidity and good solvent vapors, with the aim of monitoring (through single-chain AFM measurements) both chain swelling when PIC molecules were solvated by the good solvent and the collapse of the side chains under poor-solvent conditions [164].

Single-chain AFM was further shown to be critical in probing chemical contrast in single polymer macromolecules, including PMB, P2VP, and PS_7_-P2VP_7_ [131,165]. This contrasting method for AFM was first reported by Kiriy and coworkers in 2003, when they demonstrated a significantly enhanced resolution in single-chain AFM experiments conducted on positively charged polymeric chains adsorbed on mica or silicon wafers by depositing contrasting agents, such as negatively charged cyanide-bridged complexes or hexacyanoferrate anions [131]. Moreover, while they observed no changes in the chain conformation of the studied single polymer chains, they showed that the contrasting agents could be removed without affecting the chain conformation [131]. Later on, they extended these single-chain studies to other substrates, such as glass; however, in this case, the use of negatively charged clusters led to clear conformational distortions and possibly to chain fragmentations [165]. Note that silicon tips exhibiting a radius of 10–20 nm, a spring constant of 30 N/m, and a resonance frequency of 250–300 kHz were employed in these studies.

## 8. Other Applications of Single-Chain AFM

There are other interesting applications of single-chain AFM on polymeric systems. Because such applications are less common and there are very few examples in the literature that describe them, we cannot dedicate a separate section of this work to them. Instead, we have decided to present them together in this final section. Here, we include the use of single-chain AFM to estimate the glass transition temperature of single polymer chains [133], demonstrate the scission of polymeric chains [134], or emphasize other interactions between natural polymeric species [166].

In the first case, using AFM, Fujita and coworkers monitored the behavior of single polymer chains (isotactic and atactic PMMA, as well as P2VP) adsorbed on mica as their temperatures were incrementally increased from room temperature to 200 °C. They observed that such single chains started to move at around 100 °C for both types of PMMA and at 150 °C for P2VP, assigning these temperatures as their glass transition temperatures [133].

In the second case, Sheiko and coworkers adsorbed brush-like polymeric chains, made by attaching long poly(n-butyl acrylate) (PBA) side chains to a poly(2-hydroxyethyl methacrylate) backbone, on mica. They further demonstrated, using single-chain AFM (by employing probes displaying a resonance frequency of about 140 kHz, a spring constant of about 5 Nm, and a radius < 10 nm), that such adsorption led to conformational deformations in the brush-like chains, accompanied by the rupture of powerful carbon–carbon covalent bonds in the main chain backbone. These ruptures were attributed to the tension generated along the backbone by the attractive interactions between the long side chains and mica [134].

Finally, in the third case, Sukhanova and coworkers employed single-chain AFM to monitor various interactions between nuclear poly(ADP-ribose) polymerases (PARPs) 1 and 2 and a substrate of DNA in its undamaged and single-strand damaged forms. The authors emphasized the differences in the affinity of the two PARPs for DNA intermediates and also demonstrated the dependence of the catalytic activity of PARPs on the type of single-strand DNA damage [166].

## 9. Limitations and Challenges of Single-Chain AFM

As discussed in the previous sections, AFM imaging is a powerful technique that can be performed in various modes [167]. It is considered an essential method for studying single polymer chains and their interactions with surfaces at the nano scale, achieving sub-nanometer lateral resolutions [167,168,169] and angstrom resolutions in the vertical dimension [170]. However, despite its significant advantages, AFM also has notable limitations and challenges. These include overall tip-limited resolution; for instance, the tip structure and mechanical properties of the cantilever can significantly impact the measurement resolution and image quality [170,171]. Additional limitations encompass environmental effects, the complexity of data interpretation, lateral tip–sample drift, and often slow data acquisition times, which hinder the observation of dynamic processes or conformational transitions occurring in single polymer chains on the nano- to microsecond timescales [75]. Although advances in image acquisition speed are continuously reported [170], these challenges remain pertinent.

The aforementioned drawbacks can significantly impact experimental outcomes. For instance, one of the inherent challenges of single-chain AFM is achieving a high spatial resolution, which is essential for deciphering local conformations at the segment level. This can be accomplished by combining single-chain AFM with all-atom molecular dynamics simulations [129]. However, spatial resolution is heavily dependent on the size and sharpness of the AFM probe or cantilever, the imaging force applied, and the properties of the polymeric material itself. The size of the AFM tip can introduce convolution effects, resulting in broader images of molecular features than those that actually exist [132]. This phenomenon can lead to inaccuracies in interpreting the height and lateral dimensions of individual polymer chains, as finer details may be obscured. This issue is particularly pronounced for flexible polymers, such as proteins, which can exhibit blurriness in their AFM images due to their ability to “escape” compression by the AFM tip [172,173]. Nevertheless, the AFM technique is continuously evolving, and the dimensions of structures obtained from AFM images can often be corrected based on the tip radius [165].

Moreover, single-chain AFM imaging is sensitive to environmental conditions [164], including humidity and temperature, which can significantly impact the behavior of individual polymer chains [174]. Furthermore, conducting single-chain experiments on specific types of surfaces [107], particularly those with certain roughness, remains highly challenging [131]. It is well-established that the environment can alter the conformational state of polymers and their interactions with surfaces, potentially leading to inconsistent results across different experiments. Consequently, interpreting AFM images can be difficult and may require sophisticated models to extract meaningful information about the conformations of polymer chains. Additionally, the operational mode of AFM can introduce artifacts, especially when the tip interacts with the polymer chains. Such artifacts, which may arise from the tip’s contact force or lateral movement during imaging, can distort the perceived structure of the polymer chains. To mitigate the unavoidable tip–sample interactions in AFM analysis, surfaces that undergo special treatments may be employed [107].

Additionally, it is important to note that preparing samples for (single-chain) AFM imaging requires meticulous handling to prevent damage to or alteration of the polymer chains. Maintaining stable conditions during imaging is critical, as any drift in the system can lead to inconsistencies and complications in the acquired images. This is particularly crucial when conducting dynamic studies, such as observing conformational changes in real time, where the inherent stability of single chains during prolonged imaging may become a concern. Furthermore, not all polymer systems are suitable for single-chain imaging via AFM. For example, polymers that do not adhere well to surfaces or those that form aggregates can present challenges for effective visualization, complicating the accurate assessment of their conformation and behavior. This limitation restricts the range of materials that can be effectively studied using this technique.

Finally, some of the limitations of AFM can be further mitigated by employing complementary and faster imaging techniques, such as scanning (SEM) or transmission (TEM) electron microscopies [123], as well as optical microscopy techniques that operate beyond the diffraction limit, such as high-resolution fluorescence imaging [175]. It is important to note that although these techniques may offer improved lateral resolution, many of them can be invasive to single polymer chains. Furthermore, none of these methods provide information on height topography, making it impossible to quantify the height of a single polymer chain and, consequently, its molecular volume. 

## 10. Conclusions

In this review, we summarize the most significant scientific reports on the utilization of single-chain AFM. We conclude that this AFM technique remains a valuable complementary tool for directly observing a wide variety of isolated polymer chains deposited on solid surfaces, particularly when specific objectives are targeted and conventional characterization techniques fail or are inadequate. For example, confirming the molecular structure of highly complex polymeric chains with hyperbranched or heteroarm star-shaped configurations and multiple arm blocks can be challenging. In such cases, direct observation using single-chain AFM is essential. Similarly, the utilization of single-chain AFM when dealing with various biomacromolecules, such as plasmid DNA, xanthan, aggrecan, *β*-glucans, gum arabic, pectin, etc., is necessary. Here, single-chain AFM can either reveal the conformation of such biomolecules or their constituent monomer parts (along with the precise dimensions of the molecules, their double-strand helical structure, their molecular weight, etc.) or observe important conformational transitions to understand the collapse of polymer chains and their aggregation/coiling, fusion, folding, extension, movement, or “reptation” on a solid substrate, etc.

Furthermore, single-chain AFM significantly contributes to our understanding of processes such as polymer crystallization and self-assembly, particularly due to its ability to monitor chain folding and extension. Consequently, stem-level crystallization, the growth of unimolecular crystals into multichain crystals, and the assembly of individual chains of various biopolymers into supramolecular (fibrillar) entities can be observed in great detail using single-chain AFM. Additionally, single-chain AFM can be used not only to determine the glass transition temperature of isolated polymer chains by tracking their movements at various temperatures but also to monitor the rupture of covalent bonds in the main chain backbone and observe various cross-linking reactions. These applications of single-chain AFM are crucial for designing, developing, and integrating the studied materials into practical applications, such as biological mimics for therapeutics, unimolecular devices for drug delivery, single insulated molecular wires for molecular electronics, catalysis and organic electronics, the fabrication of effective emulsifiers and stabilizers in oil-in-water emulsions, and the production of self-assembled clusters for innovative electrooptic materials, among others.

Finally, it is important to note that while single-chain AFM imaging offers invaluable insights into the behavior of individual polymer chains, it also presents several limitations. These include challenges related to tip-dependent resolution, environmental sensitivity, complex data interpretation, tip artifacts, sample preparation and stability, and material applicability. Researchers must carefully consider these hurdles and work towards overcoming them by continuously advancing AFM technology. This can be achieved through the design of new ultra-fine, long-lasting tips, the development of improved material processing tools, the creation of more suitable surfaces, and the enhancement of data analysis techniques, as well as the establishment of more consistent statistical models for polymer chains and conformational analysis.

## Figures and Tables

**Table 1:** Summary of the benefits that can be obtained when employing the AFM technique to image single polymer chains of various architectures and chemical structures.

Polymer System	Benefits of Single-Chain AFM Conducted on Isolated Polymer Chains	Ref.
Plasmid DNA	First visualization of a conformation adopted by a single DNA chain	[108]
PS-*b*-PMMA	First visualization of a conformation of a single synthetic polymer chain	[109]
Xanthan polysaccharide	Demonstration of a double-stranded structure adopted by a xanthan helix	[110]
PBPEM-*g*-(P*n*BuA-*b*-PS) BCP	Visualization of BCPs adopting extended molecular conformations, along with their side chains	[82]
DNA	Precise determination of the length of a DNA molecule	[83]
PBA brushes	Accurate measurement of the molecular weight of a molecule	[84]
Cartilage aggrecan macromolecules	Visualization of a conformation adopted by individual monomers and their constituent parts; determination of the end-to-end length	[85]
Oat *β*-glucans	Exact determination of the contour length, end-to-end distance, persistence length, and *M_w_*	[86]
PS_7_-P2VP_7_ heteroarm star copolymer	Confirming the number of P2VP arms and visualization of the unimolecular micellar structure	[89]
Polystyrene- and poly(butyl acrylate)-based hybrid BCPs	Validating the generation of hybrid BCPs and visualization of a single nanoparticle–coil copolymer architecture	[87]
Arborescent PS	Observing the molecular structure of tree-like high-molecular-weight PSs and validating the mechanism of inimer polymerization	[90]
B-SHBPs	Visualization of the molecular structure of various SHBPs	[96]
Dendronized PFSs	Observing unimolecular spherical cocoons and other elongated structures comprising single PFS chains	[91]
Dendronized polynorbornenes	Detecting unimolecular tadpoles	[92]
	Visualization of single random coil and rigid rod structures	[94]
Dendronized conjugated di-BCPs	Deciphering the structure of single molecular wires comprising a regioregular backbone surrounded by bulky dendrons	[93]
Gum arabic and soybean polysaccharides	Validating a previous structural model assuming the existence of long sugar side chains on the main backbone; differentiating the linear and branched appearances of gum arabic and soybean polysaccharides	[98]
Pectin heteropolysaccharide	Confirming the branched appearance of polysaccharides and the minimal impact of the loss of neutral sugars on the size or the branching density of the individual chains	[99]
Polysaccharide rhamnogalacturonan I	Demonstrating the existence of regular side chains on isolated polysaccharide molecules	[100]
PCEVE-based polymers	Observing the linear, cyclic, and tadpole-shaped dimer molecules consisting of a ring and a linear chain, trefoil knot rings, and figure-of-eight dimer rings, as well as catenane molecular structures	[103]
Grafted polymers based on selenol moieties	Monitoring the collapse of single polymer chains containing selenol moieties	[104]
Polymer brushes	Visualization of molecular cycles made of cyclic polymers and their fusions into multimers	[105]
Polysilane	Observing dots of globular conformation and ropes with rigid rod conformations	[111]
PS-*b*-PMMA	Visualizing the transition from single non-aggregated PMMA chains to aggregated PMMA chains, generating a condensed monolayer, to PMMA chains adopting an expanded coil conformation	[112]
Carrageenan polysaccharides	Visualization of a coil to helix transition in an iota-Na-carrageenan single chain on mica and demonstrating the intramolecular generation of a unimeric helix from such a chain	[113]
DNA	Monitoring the dynamic motions of DNA single molecules and their structural changes within DNA origami surface relief structures	[107]
PMB	Revealing multiple conformational changes from a coil to a pearl necklace–globule, to a globule of single PMB chains on mica	[114]
*it*-PMMA	Demonstrating the “reptational” movements of PMMA flexible chains along their chain axis on mica	[115]
	Observing, for the first time, the crystallization behavior of a single polymeric chain at the molecular level	[116]
	Demonstrating the folding of isolated *it*-PMMA chains upon their crystallization	[117]
	Monitoring the growth of crystals from unimolecular to multichain structures	[118]
	Visualizing the stem-level crystallization of a single *it*-PMMA chain into a folded-chain crystal	[119]
Collagen, *k*-carrageenan, xanthan, gellan, scleroglucan	Monitoring the assembly of individual chains of various biopolymers into supramolecular (fibrillar) entities	[120]
Cellulose	Demonstrating the transition from aligned single chains to globular aggregates	[121]
Tannin/TMV	Studying the effect of tannin on the aggregation of TMV particles and its antiviral mechanism	[122]
PCPP/antigenic proteins	Revealing the ability of antigenic proteins to bind at single PCPP chains to generate single-chain compact spherical complexes or stiffened coils	[123]
Polysaccharide	Observing the motion of a polysaccharide under an aqueous buffer solution	[124]
P2VP	Watching the transition from extended coils to compressed globules of adsorbed P2VP single chains	[125]
PMB	Seeing the transition from extended coils to pearl necklace-like globules of adsorbed PMB single chains	[126]
PSS	Revealing how PSS single chains adsorbed on mica are adopting a wormlike coil conformation	[127]
Nafion	Showing how autoclaved Nafion single chains adsorbed on mica and graphite adopt a conformation resembling compact globules	[128]
PMMA	Monitoring the rigidification of single PMMA chains with temperature increases	[129]
DNA	Observing the transition from 2D random coils to stretched DNA conformations	[130]
PMB, P2VP, PS_7_-P2VP_7_	Demonstrating the first enhancement of resolution of single-chain AFM on positively charged polymeric chains by using contrasting agents	[131]
Alginate, circular plasmid DNA, acetan, xanthan	Showing that the complexation of polyanions with chitosan generates torus-like morphologies	[132]
Isotactic and atactic PMMA, P2VP	Determining the glass transition temperature of isolated polymer chains by monitoring their movements at various temperatures	[133]
PBA-based brushes	Monitoring the rupture of covalent bonds in the main chain backbone	[134]
Polycarbonate	Monitoring the cross-linking reactions that generate SCPNPs	[135]
UPy-functionalized PMMA	Observing the chain folding upon the formation of individual SCPNPs	[136]

## Data Availability

Not applicable.
